# Mechanisms by Which Obesity Promotes Acute Graft-*Versus*-Host Disease in Mice

**DOI:** 10.3389/fimmu.2021.752484

**Published:** 2021-10-11

**Authors:** Lam T. Khuat, Logan V. Vick, Eunju Choi, Cordelia Dunai, Alexander A. Merleev, Emanual Maverakis, Bruce R. Blazar, Arta M. Monjazeb, William J. Murphy

**Affiliations:** ^1^ Department of Dermatology, School of Medicine, University of California, Davis, Sacramento, CA, United States; ^2^ Department of Pathology, Microbiology and Immunology, School of Veterinary Medicine, University of California, Davis, Davis, CA, United States; ^3^ Masonic Cancer Center and Division of Blood & Marrow Transplant & Cellular Therapy, Department of Pediatrics, University of Minnesota, Minneapolis, MN, United States; ^4^ Department of Radiation Oncology, School of Medicine, University of California, Davis, Sacramento, CA, United States; ^5^ Department of Internal Medicine, School of Medicine, University of California, Davis, Sacramento, CA, United States

**Keywords:** obesity, microbiome, GVHD, high-fat (HF) diet, cytokine storm

## Abstract

The efficacy of allogeneic hematopoietic stem cell transplantation (allo-HSCT) is limited by the occurrence of acute and chronic graft-*versus*-host disease (GVHD). We have recently demonstrated that obesity results in exacerbated acute gastrointestinal GVHD in both mouse models and clinical outcomes due to increased pro-inflammatory cytokine responses and microbiota alterations. We therefore wanted to delineate the role of the various parameters in obesity, adiposity, effects of high-fat (HF) diet, and the role of microbiome on GVHD pathogenesis, by taking advantage of a mouse strain resistant to diet-induced obesity (DIO). Female BALB/c mice are resistant to DIO phenotype with approximately 50% becoming DIO under HF diets. The DIO-susceptible recipients rapidly succumb to acute gut GVHD, whereas the DIO-resistant recipient littermates, which do not become obese, are partially protected from GVHD, indicating that being on HF diet alone contributes to but is not the primary driver of GVHD. Microbiome assessment revealed restricted diversity in both cohorts of mice, but coprophagy normalizes the microbiota in mice housed together. We then individually housed DIO-resistant, DIO-susceptible, and lean control mice. Notably, each of the individually housed groups demonstrates marked restricted diversity that has been shown to occur from the stress of single housing. Despite the restricted microbiome diversity, the GVHD pathogenesis profile remains consistent in the group-housed mice, with the lean control single-housed mice exhibiting no acute GVHD and DIO-resistant recipients showing again partial protection. These results demonstrate that the deleterious effects of obesity on acute gut GVHD are critically dependent on adiposity with the HF diet also playing a lesser role, and the microbiome alterations with obesity instead appear to fuel ongoing acute GVHD processes.

## Introduction

Allogeneic hematopoietic stem cell transplantation (allo-HSCT) is used for the treatment of a variety of hematopoietic disorders ranging from cancer to aplastic anemia. However, the key limitation to allo-HSCT is the development of graft-*versus*-host-disease (GVHD) that can manifest through either acute or chronic pathologies resulting in significant morbidity. A hallmark of acute GVHD is that of an inflammatory cascade resulting in a “cytokine storm” or pro-inflammatory cytokines attack multiple organs including the gastrointestinal (GI) tract, liver, lung, and skin, with chronic GVHD being more delayed and fibrotic in nature. The acute GVHD process driven by alloreactive donor T cells is fueled in part by the cytoreductive conditioning applied that causes tissue damage to susceptible organs (i.e., the GI system) and pro-inflammatory cytokine induction. Different mouse models can mirror these pathologic processes and can be highly selective depending on the strain combination for modeling acute or chronic GVHD.

Obesity, which is characterized as body mass index (BMI) greater than 30, is known to modulate immune responses and induce a meta-inflammatory state that has been linked to worse outcomes in various disease states (1, 2). Due to its rising prevalence in the US population, understanding the effect of obesity on health outcomes is critical. We have observed that obesity exacerbates immune dysregulation in mice and promotes a GVHD-like lethal pro-inflammatory cytokine storm following strong systemic immunotherapy or lipopolysaccharide (LPS) injection (3). In the context of allo-HSCT, we have recently demonstrated that mice with diet-induced obesity (DIO) placed on high-fat (HF) diets developed more severe acute GVHD due in part to the induction of a heightened pro-inflammatory cytokine storm (4). These effects were also observed clinically in high-BMI HSCT recipients (4–7). Interestingly, it was also observed that obesity resulted in restricted microbiome diversity and increased gut permeability, which may account for the specific targeting of the gut for acute GVHD attack (4, 8–10). Prophylactic treatment with antibiotics could partially protect the DIO mice from acute GVHD, indicating that the microbiome likely plays a role (4). The microbiome has been under intensive study in HSCT, particularly clinically, with outcomes being linked to specific deleterious and beneficial bacteria species (4, 11–14). Also, the reduction of microbiome diversity has been previously reported to be a negative prognostic indicator following HSCT (11, 14). However, with the DIO mouse model, it was not clear whether the microbiome alterations were primarily responsible for the increased GVHD observed. With HF diets, there is markedly increased adiposity that may be equally as important as immunomodulators fueling pro-inflammatory cytokine responses (3), but there is evidence that the diet itself may also affect immune and in particular T-cell responses due to free fatty acid metabolism (15, 16). We therefore wanted to delineate in obesity which parameters were the major drivers in the augmented acute gut GVHD observed.

Here, we attempt to discern the individual contributions of adiposity, diet, and microbiome on obesity-associated poor GVHD outcome. For these studies, the BALB/c DIO mouse model was chosen because when placed on an HF diet, only half of the female mice become obese even after several months of exposure (17, 18). The other half of the mice, which do not become obese, have weights similar to control mice fed a low-fat diet. This provides a unique opportunity to delineate the individual roles of obesity based on weight and diet on obesity-associated poor GVHD outcome. Using a major histocompatibility complex (MHC)-matched but minor histocompatibility antigen (mHA) mismatch strain combination where normally only later chronic GVHD occurs (19, 20), we observed that DIO-susceptible (DIO-S) female mice succumb with nearly 100% lethality to rapid acute gut GVHD, whereas the normal-weight DIO-resistant (DIO-R) female littermates fed the same diet also displayed increased acute GVHD but had improved outcomes, with more than 50% of mice recovering but later developing similar chronic GVHD as the lean recipients. As both co-housed DIO-S and DIO-R mice had similar microbiome profiles and decreased microbiota diversity when compared to lean controls, we then wanted to assess the role of the microbiome by single housing the mice on the different diets. Surprisingly, single cage housing resulted in marked microbiome reductions in diversity in all cohorts. After allo-HSCT in singly housed mice, the patterns of acute GVHD susceptibility remained the same as the group-housed recipients (with no GVHD in the lean recipients and comparable GVHD outcomes in the DIO-S and DIO-R cohorts), indicating that reduction of microbiome diversity alone was not sufficient to drive acute GVHD susceptibility. These data highlight the dynamics between obesity, diet, and microbiome and demonstrate that adiposity is the major driver for increased gut GVHD, although high-fat (HF) diet exposure can play a role to a lesser extent.

## Methods

### Mice and Allo-HSCT

In this study, 6–8-week-old female BALB/c and C57BL/6 mice were obtained from Taconic Farms. Mice were placed on an HF diet or low-fat diet (D12492 or D12450J, Research Diets, Inc.). In our singly housed mouse model, 6- to 8-week-old female BALB/c mice were placed in individual cages with low-fat or HF diet for at least 4 months to establish their body weight and gain phenotype before being used for HSCT.

The 8–10-week-old female B10.D2 mice were obtained from Jackson Laboratory and were used as donor mice. In order to create the MHC matched, mHA GVHD model, BALB/c mice (H2^d^) received lethal total body irradiation (TBI) (800 cGy; ^137^Cs source) and underwent transplantation with bone marrow cells with or without splenocytes (25 × 10^6^) from the donor B10.D2 mice (H2^d^) as described previously (4, 19). All mice were maintained at the University of California (UC), Davis Medical Center’s vivarium in accordance with Institutional Animal Care and Use Committee (IACUC) standards.

### Acute GVHD and Chronic GVHD Clinical Score Criteria

Acute GVHD clinical scores were determined based on weight loss (0–2), hunching (0–2), diarrhea (0–2), fur texture (0–2), and skin integrity (0–2). Mice were euthanized if they had a total score over 7 out of 10 or showed severe hunching (4, 21).

For sclerodermatous chronic GVHD, BALB/c mice were monitored for skin clinical scores and body weight loss post-allo-HSCT as described previously (4, 20). Briefly, skin clinical scores were assigned as follows: 0, healthy appearance; 1, skin lesions with alopecia area <1 cm^2^; 2, skin lesions with alopecia area of 1–2 cm^2^; 3, skin lesions with alopecia area >2 cm^2^. Tail, ear, or paw scaling represented an additional 0.3 point for each lesion. Mice with a clinical skin score >3.3 (on a scale of 0–3.9) or with severe ischemic skin and tail lesions and hunching were euthanized per guidelines.

### Magnetic Resonance Imaging

Mice were anesthetized with isoflurane and oxygen and then scanned on a Biospec 70/30 7.0-Tesla small-animal magnetic resonance imaging (MRI) system (Bruker Biospin Inc.) using a 60-mm quadrature transmitter/receiver coil for whole-body imaging. The scanning protocol consisted of the multislice with multi-echo spin-echo sequence with a single echo and with respiratory gating to minimize breathing artifacts. Scan parameters were echo time (TE) of 7.062 and repetition time (TR) of 775, conducted with and without chemical-selective fat suppression. Slice images were obtained in the coronal direction to improve spatial resolution while keeping scan time and TR at minimum. The in-plane matrix was 200 × 267 with a resolution of 0.3 × 0.3 mm. Forty-four slices were acquired with a slice thickness of 0.6 mm. Field of view was 6 cm × 8 cm × 2.64 cm. Difference images were generated by subtracting the fat-suppressed images from the non–fat-suppressed images to identify the three-dimensional distribution of fat deposits. Physiological monitoring (temperature and respiration) was used during the entire scan to ensure consistency and animal physiological stability.

### Histology and Histopathology Scores

Tissues harvested from mice were placed in 10% formalin, embedded in paraffin, sectioned, and stained with hematoxylin and eosin. Tissue sections were evaluated and graded by a board-certified veterinary pathologist in a single-blinded fashion. GI pathology was scored on a scale of 0–3 based on goblet cell loss, gland epithelial piling, and karyomegaly. Images were visualized with a Vanox AHBS3 microscope with an SPlan Apo 20×/0.70 NA objective (Olympus, NY, USA). Images were acquired with a SPOT RT color digital camera using SPOT version 4.0.2 software (Diagnostic Instruments, MI, USA).

Fibrotic skin samples were assessed by Masson’s trichrome staining with a Masson’s 2000 Trichrome kit (SKU# KTMTR2; American MasterTech, Lodi, CA, USA). Images were visualized with a Vanox AHBS3 microscope with an SPlan Apo 20×/0.70 numerical aperture (NA) objective (Olympus, NY, USA) and acquired with a SPOT RT color digital camera using SPOT version 4.0.2 software (Diagnostic Instruments, MI, USA).

### Cytometric Bead Array

Serum cytokines were measured by cytometric bead array (CBA) flex set kits (BD Biosciences, San Jose, CA): mouse tumor necrosis factor (TNF) (Cat# 558299), mouse interleukin (IL)-6 (Cat# 558301). Serum samples were diluted 1:4 using assay diluent solution provided in the kit. Capture beads and detection beads were added as described in the user guide. Cytokine concentration was measured by flow cytometry.

### Antibodies and Flow Cytometry Analysis

Single-cell suspensions (1 million cells) were first incubated with Fc Block (BD Pharmingen, San Diego, CA, USA) for 10 min and then coincubated with antibodies for 20 min at 4°C, followed by washing with staining buffer (phosphate-buffered saline + 1% fetal bovine serum). Flow cytometry analysis was performed with the LSR Fortessa cell analyzer (BD Biosciences, San Jose, CA, USA), and data were analyzed using FlowJo v10 software (FlowJo, Ashland, OR, USA). We used the following fluorochrome-conjugated monoclonal antibodies purchased from BioLegend (San Diego, CA, USA): CD45–Pacific Blue (30-F11), CD19–Brilliant Violet 785 (6D5), CD11c–phycoerythrin (PE)/Cy7 (N418), I-A/I-E–APC (allophycocyamin)/Cy7 (M5/114.15.2). We used the following fluorochrome-conjugated monoclonal antibodies purchased from BD Biosciences (San Jose, CA, USA): CD229.1-PE (30C7).

### Mouse Microbiome Analysis

Microbiome data are uploaded into a biorepository with Bioproject ID PRJNA758120. DNA was isolated using the Qiagen DNeasy PowerSoil kit (Qiagen) with the following modifications. After addition of buffer C1, samples were incubated at 65°C for 10 min and subjected to homogenization using a Biospec Mini-Beadbeater (Biospec Products) for 2 min. An additional wash step with 100% ethanol was included preceding the wash with kit buffer C5. Samples were eluted in 100 μl of buffer C6. Primers 319F (**TCGTCGGCAGCGTCAGATGTGTATAAGAGACAG**(spacer)*GT* ACTCCTACGGGAGGCAGCAGT) and 806R (**GTCTCGTGGGCTCGGAGATGTGTATAAGAGACAG**(spacer)*CC* GGACTACNVGGGTWTCTAAT) were used to amplify the V3–V4 domain of the 16S rRNA using a two-step PCR procedure. In step 1 of the amplification procedure, both forward and reverse primers contained an Illumina tag sequence (bold), a variable-length spacer (no spacer, C, TC, or ATC for 319F; no spacer, G, TG, or ATG for 806R) to increase diversity and improve the quality of the sequencing run, a linker sequence (italicized), and the 16S target sequence (underlined). Each 25-μl PCR reaction contained 1 Unit Kapa2G Robust Hot Start Polymerase (Kapa Biosystems), 1.5 mM MgCl_2_, 0.2 mM final concentration dNTP mix, 0.2 μM final concentration of each primer, and 1 μl of DNA for each sample. PCR conditions were as follows: an initial incubation at 95°C for 3 min, followed by 25 cycles of 95°C for 45 s, 50°C for 30 s, 72°C for 30 s, and a final extension of 72°C for 3 min. In step 2, each sample was barcoded with a unique forward and reverse barcode combination using forward primers (**AATGATACGGCGACCACCGAGATCTACAC**NNNNNNNNTCGTCGGCAGCGTC) with an Illumina P5 adapter sequence (bold), a unique 8-nt barcode (N), a partial matching sequence of the forward adapter used in step 1 (underlined), and reverse primers (**CAAGCAGAAGACGGCATACGAGAT**NNNNNNNNGTCTCGTGGGCTCGG) with an Illumina P7 adapter sequence (bold), unique 8-nt barcode (N), and a partial matching sequence of the reverse adapter used in step 1 (underlined). The PCR reaction in step two contained 1 Unit Kapa2G Robust Hot Start Polymerase (Kapa Biosystems), 1.5 mM MgCl_2_, 0.2 mM final concentration dNTP mix, 0.2 μM final concentration of each uniquely barcoded primer, and 1 μl of the product from the PCR reaction in step 1 diluted at a 10:1 ratio in water. PCR conditions were as follows: an initial incubation at 95°C for 3 min, followed by eight cycles of 95°C for 30 s, 58°C for 30 s, 72°C for 30 s, and a final extension of 72°C for 3 min. The final product was quantified on the Qubit instrument using the Qubit Broad Range DNA kit (Invitrogen), and individual amplicons were pooled in equal concentrations. The pooled library was cleaned utilizing Ampure XP beads (Beckman Coulter), then the band of interest was further subjected to isolation *via* gel electrophoresis on a 1.5% Blue Pippin HT gel (Sage Science). The library was quantified *via* qPCR followed by 300-bp paired-end sequencing using an Illumina MiSeq instrument (Illumina) in the Genome Center DNA Technologies Core, University of California, Davis. DNA extractions and library preparation were performed by the UC Davis Host Microbe Systems Biology Core Facility.

Heatmaps were generated with R package “pheatmap”. Boxplots and volcano plots were created with R. The linear discriminate effect size (LefSe) program was used to ascertain any significant differences in taxonomic abundance (22). The LefSe program uses the Kruskal–Wallis sum-rank test to detect taxa with significant differential abundance in relation to class, and then biological significance is determined by pairwise tests between subclasses using the Wilcoxon rank-sum test. Finally, linear discriminate analysis is used to estimate the effect size of each differentially abundant taxa, and taxanomic cladograms were generated to highlight significant differences in taxa.

### Statistical Analysis

Acute GVHD clinical scores and skin clinical scores were analyzed by two-way analysis of variance (ANOVA) with Tukey’s *post-hoc* test for comparison among groups. Flow cytometry data were analyzed using the Student’s *t*-test. A p-value <0.05 was considered significant. Survival curves were plotted on a Kaplan–Meier curve and analyzed by a log-rank test. The data were graphed and statistically analyzed using GraphPad Prism (GraphPad Software, Inc., CA, USA).

## Results

### High Body Weight, Not High-Fat Diet Exposure, Is the Major Driver of Lethal GVHD Post-HSCT in Mice

**Figure 1:**
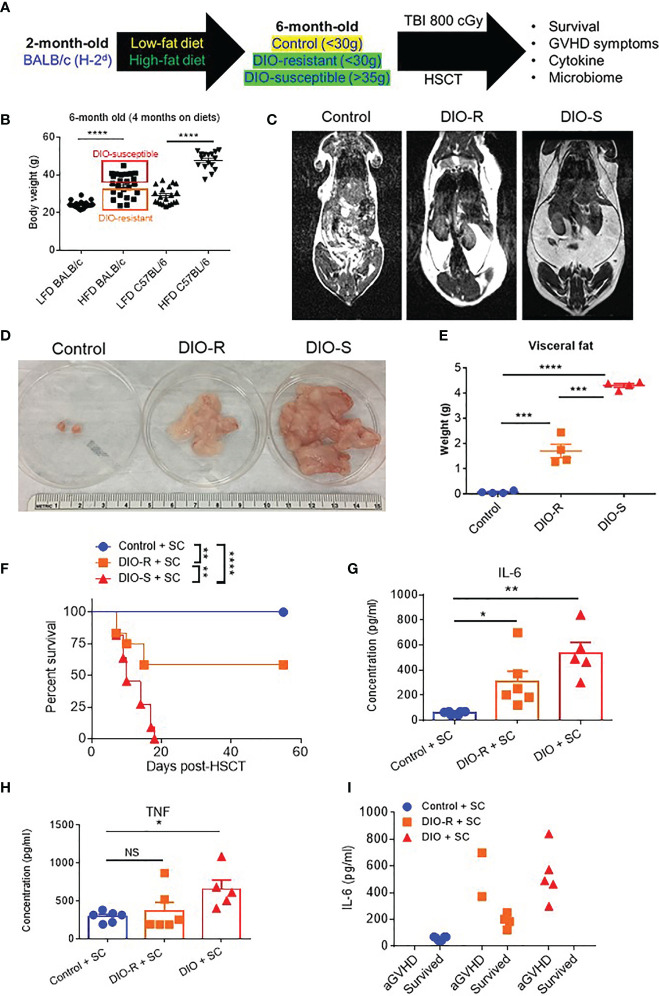
**(A)** Lethally irradiated control, DIO-R, and DIO-S BALB/c mice received 8 million bone marrow cells and 25 million splenocytes from donor B10.D2 mice. **(B)** Body weights of BALB/c and C57BL/6 mice after 4 months on LF or HF diet (n=16-24/group). **(C)** Magnetic resonance imaging scans of control, DIO-R, and DIO-S mice. Fat tissue is white. **(D)** Representative images of visceral fat content of control, DIO-R, and DIO-S mice. **(E)** Quantification of visceral fat content of control, DIO-R, and DIO-S mice (n=4/group). **(F)** Survival rate post-HSCT (n=12/group). **(G)** Serum IL-6 and **(H)** TNF concentrations at day 7 post-HSCT (n=5-6/group). **(I)** Correlation between acute GVHD outcomes and IL-6 levels (n=5-6/group). Graphs depict mean ±s.e.m. Survival curve **(F)** was plotted on a Kaplan-Meier curve and analyzed by a log-rank test. One-way ANOVA test was used in **(B, F, G)**. *p < 0.05, **p < 0.01, ***p < 0.001, ****p < 0.0001, NS, not significant.

**Figure 2:**
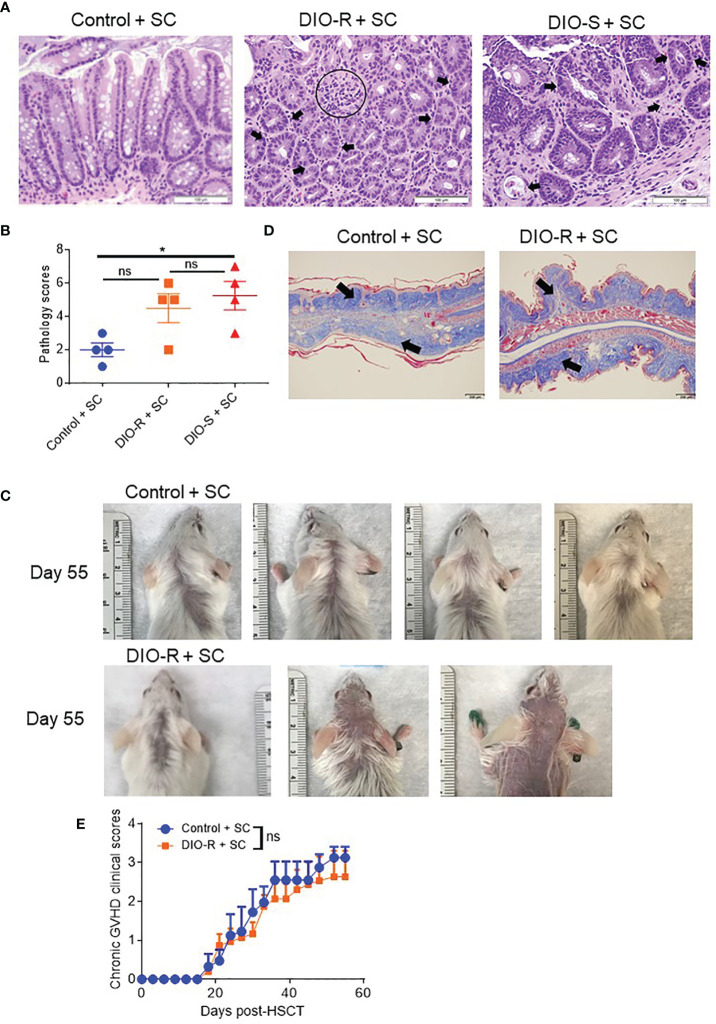
**(A)** Representative images of H&E staining from colon samples at day 7 post-HSCT. The scale bar is 100μm. Circle indicates severe goblet cell loss and mild, multifocal lamina proprial inflammation. Arrows indicate piling of glandular epithelial cells, degenerate crypts, apoptotic crypt abscess and rare karyomegalic cells. **(B)** Pathology scores of samples from **(A)** (n=4/group). **(C)** Representative images of sclerodermatous GVHD with alopecia at day 55 post-HSCT. **(D)** Representative photos of sclerodermatous GVHD with tissue fibrosis by Trichrome staining. The scale bar is 200μm. Arrows indicate collagen deposition (blue). **(E)** Chronic GVHD clinical scores post-HSCT (n=3-4/group). Bar graphs depict mean ±s.e.m. One-way ANOVA test was used in **(B)**. Unpaired Student’s t test was used in **(E)**. *p < 0.05, NS, not significant.

BALB/c mice have been shown to be relatively resistant to HF diet weight gain, with some gaining weight (DIO-S) and some maintaining weight values comparable to mice on control diets (DIO-R) in the same cage (17). After 4 months on HF diet, we also observed that there was a weight range only in BALB/c mice that could be stratified into two cohorts: DIO-S [mean body weights larger than the standard deviation (SD) of body weights of control mice by 5 times, body weights are at least 35 g] and DIO-R (less than 3× SD of control mice mean body weights, lower than 30 g; **Figures 1A, B**; **Supplemental Figure S1A**). Body weight gain kinetics also showed a significant difference between DIO-S and DIO-R mice (**Supplemental Figure S1B**). MRI scan and visceral fat quantification also revealed high body fat content in DIO-S mice compared to DIO-R or control mice (**Figures 1C–E**). These mice were then divided into cohorts to assess effects on GVHD outcome compared to mice on the control diet. We used the well-established MHC-matched but minor mismatch strain combination model of B10.D2 (H-2^d^) bone marrow cells and splenocytes into lethally irradiated BALB/c mice (H-2^d^) that normally results in a later sclerodermatous chronic GVHD (23, 24). However, using DIO recipients, we observed that obesity triggered a rapidly lethal acute GVHD targeting GI tract in this strain combination along with other mouse GVHD models (4). Now, comparing DIO-S and DIO-R mice of the same strain, we observed that the DIO-S mice again all significantly succumbed to lethal GVHD, while all the lean control mice survived (**Figure 1F**). The DIO-R mice with comparable body weights as the control lean mice all showed acute GVHD symptoms with decreased survival but to a much lesser extent as the DIO-S mice (**Figure 1F**). Clinical symptoms of the DIO-S and DIO-R mice also showed that they developed acute GVHD with diarrhea, body weight loss, ruffled fur, and severe hunching (**Supplemental Figures S1C, D**). Cytokine assessment confirmed evidence of heightened TNF and significantly increased IL-6 in the serum of DIO-R and DIO-S mice early after HSCT (**Figures 1G, H** **)**. Notably, high pro-inflammatory cytokine levels of IL-6 correlate with decreased acute GVHD survival in the mice (**Figure 1I**). Based on the clinical symptoms, we examined the GI tract by histopathology and observed marked pathology affecting the small intestine and the colon of the DIO-S mice and DIO-R mice posttransplant with goblet cell loss, multifocal lamina propria inflammation (**Figures 2A, B** **)** in agreement with previous results (4). Gene expression assessment revealed a significant increase of pro-apoptotic gene Caspase 3 in the gut of DIO-S mice (**Supplemental Figure S1E**). Activated dendritic cells (MHC II^+^ CD11c^+^) were also significantly higher in the mesenteric lymph nodes of DIO-S mice (**Supplemental Figures S2A, B**). Interestingly, the surviving DIO-R and control recipients later developed typical sclerodermatous GVHD associated with this model (**Figures 2C–E**). Taken together, these results demonstrate that obesity correlates with a paradigm shift in GVHD pathogenesis, shifting a typical chronic GVHD into a lethal acute GI tract GVHD in an MHC-matched, mHA-mismatched model and that body weight and not diet appears to be the major driver correlating with GVHD outcome.

### High-Fat Diet Exposure Results in Less Diverse Microbiota

**Figure 3:**
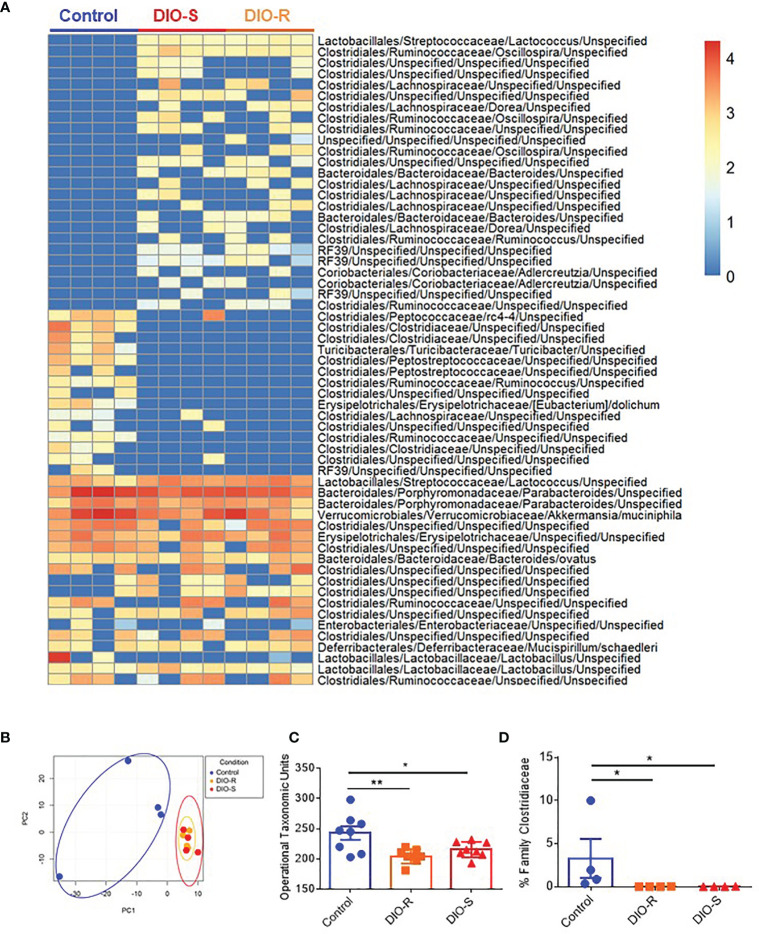
**(A)** Taxonomic profiles of resting control, DIO-R, and DIO-S BALB/c mice (n=4/group). **(B)** Principle component analysis (PCA) of microbiome profiles of control, DIO-R, and DIO-S BALB/c mice (n=4/group). **(C)** Operational taxonomic units (OTUs) in control, DIO-R, and DIO-S BALB/c mice (n=8/group). **(D)** Abundance of family Clostridiaceae of control, DIO-R, and DIO-S BALB/c mice. Bar graphs depict mean ±s.e.m. One-way ANOVA test was used in **(C, D)**. *p < 0.05, **p < 0.01.

We and others have observed that obesity induces microbiome changes in both mice and humans, resulting in marked decreases in microbial diversity that can correlate with poorer prognoses in allo-HSCT (4, 8, 9, 25). We next assessed the role of HF diet consumption on microbiome alterations in DIO-R and DIO-S cohorts. Indeed, microbiome profiles of both DIO-R and DIO-S mice shared a high level of similarity at multiple taxonomic units (**Figure 3A**). Principle component analysis showed closely distributed microbiome profiles of DIO-R and DIO-S mice (**Figure 3B**). Resting DIO-R and DIO-S mice displayed a less-diverse microbiome compared to the control (**Figure 3C**) that could be correlated with poor HSCT outcome compared to control diet recipients (4, 11, 14). Interestingly, the family *Clostridiaceae* abundance, which has been shown to correlate with better GVHD outcomes (13), was reduced in both DIO-R and DIO-S mice compared to the control mice (**Figure 3D**). These results indicated that exposure to HF diet alone regardless of body weight or adiposity can modify the gut microbiota and potentially contribute to acute GVHD pathogenesis post-HSCT but, given the comparable microbiome profiles yet different GVHD severities, was not the dominant driver in outcome.

### Singly Housed Mice Reproduce the Obesity-Resistant Phenotype and GVHD Outcomes Observed in Co-Housed Mice

**Figure 4:**
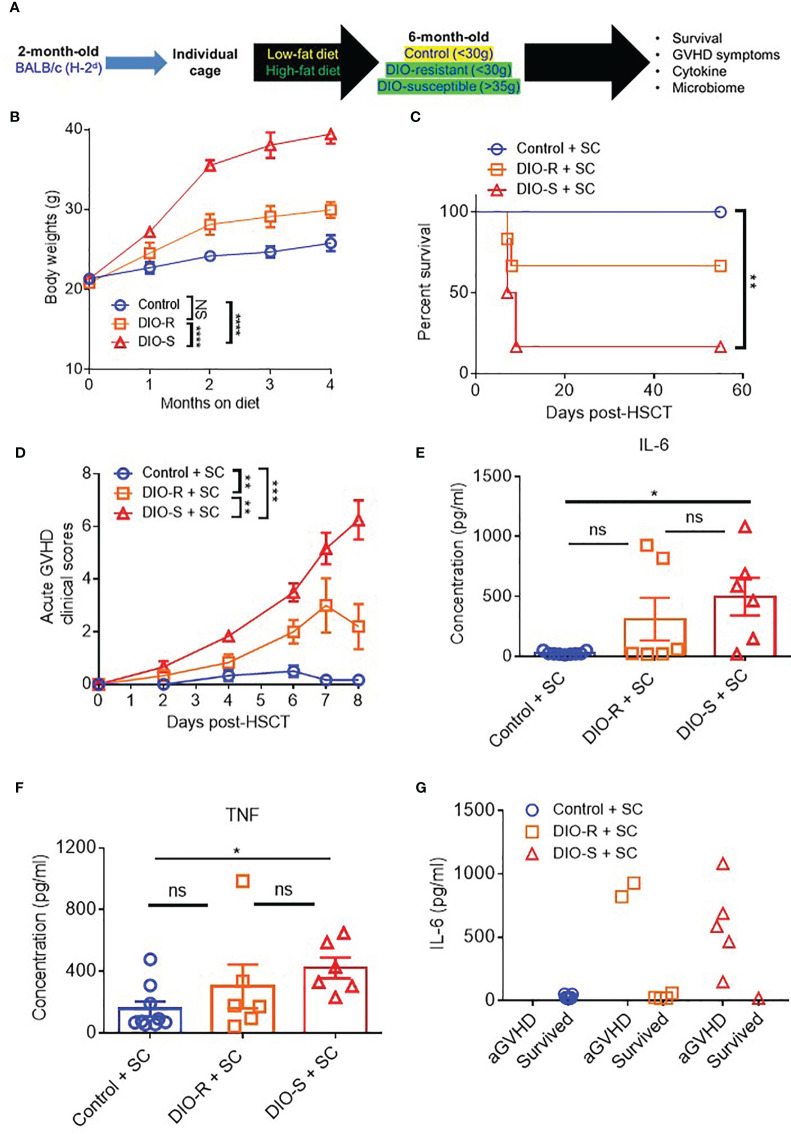
**(A)** 8-week-old BALB/c mice were individually housed and put on LF or HF diet for 4 months. Mice were then lethally irradiated and received 8 million bone marrow cells and 25 million splenocytes from donor B10.D2 mice. **(B)** Kinetics of body weight gain of BALB/c mice on LF or HF diet. **(C)** Survival rate post-HSCT (n=6-9/group). **(D)** aGVHD clinical scores post-HSCT (n=6-9/group). **(E)** Serum IL-6 and **(F)** TNF concentrations at day 7 post-HSCT (n=6-9/group). **(G)** Correlation between acute GVHD outcomes and IL-6 levels (n=6-9/group). Bar graphs depict mean ±s.e.m. Survival curve **(C)** was plotted on a Kaplan-Meier curve and analyzed by a log-rank test. Body weght curve **(B)** and clinical scores **(D)** were analyzed by 2-way analysis of variance (ANOVA) with Tukey's post hoc test for comparison among groups. One-way ANOVA test was used in **(E, F)**. *p < 0.05, **p < 0.01, ***p < 0.001, NS, not significant.

**Figure 5:**
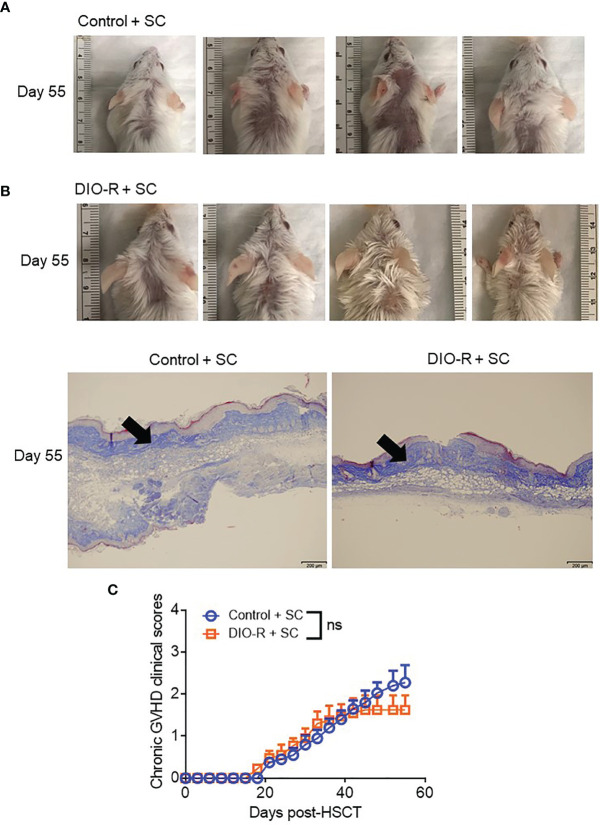
**(A)** Representative images of sclerodermatous GVHD with alopecia at day 55 post-HSCT. **(B)** Representative photos of sclerodermatous GVHD with tissue fibrosis by Trichrome staining. The scale bar is 200μm. Arrows indicate collagen deposition (blue). **(C)** Chronic GVHD clinical scores post-HSCT (n=4/group). Bar graphs depict mean ±s.e.m. Unpaired Student’s t test was used in **(C)**. NS, not significant.

Given that our data were obtained from co-housed mice (in each cage, there are both DIO-S and DIO-R mice) and that due to the coprophagy of mice that has been reported to normalize a microbiome within a cage (26), we wanted to address if singly housing the BALB/c mice prior to placing on the diets would allow for better representation of the potential microbiome alterations that can occur with HF diet and weight gain and if social hierarchy status influenced body weight gain on HF diets (**Figure 4A**). Mice were singly housed at 6–8 weeks old and given control or HF diets, with their body weights monitored. Surprisingly, singly housed BALB/c mice fed with HF diet still displayed the same obesity-resistant and obesity-susceptible phenotypes, indicating that social hierarchy and social status do not impact weight gain (**Figure 4B**). Following allo-HSCT, the patterns of acute GVHD onset also remained the same, as the singly housed DIO-S and DIO-R mice had similar outcomes compared to group-housed littermates, with the DIO-S having higher acute GVHD clinical scores and lower survival compared to DIO-R (**Figures 4C, D** **)**. These GVHD outcomes were again correlated with IL-6 and TNF levels (**Figures 4E–G**). Similarly, the lean control mice and surviving singly housed DIO-R mice developed sclerodermatous GVHD symptoms with alopecia later at 8 weeks after HSCT (**Figures 5A–C**). These results indicate that body weight gain is the primary driver in acute gut GVHD pathology following allo-HSCT.

### Singly Housing Normalizes Microbiome Profiles Between High-Fat and Low-Fat Diet-Fed Mice

**Figure 6:**
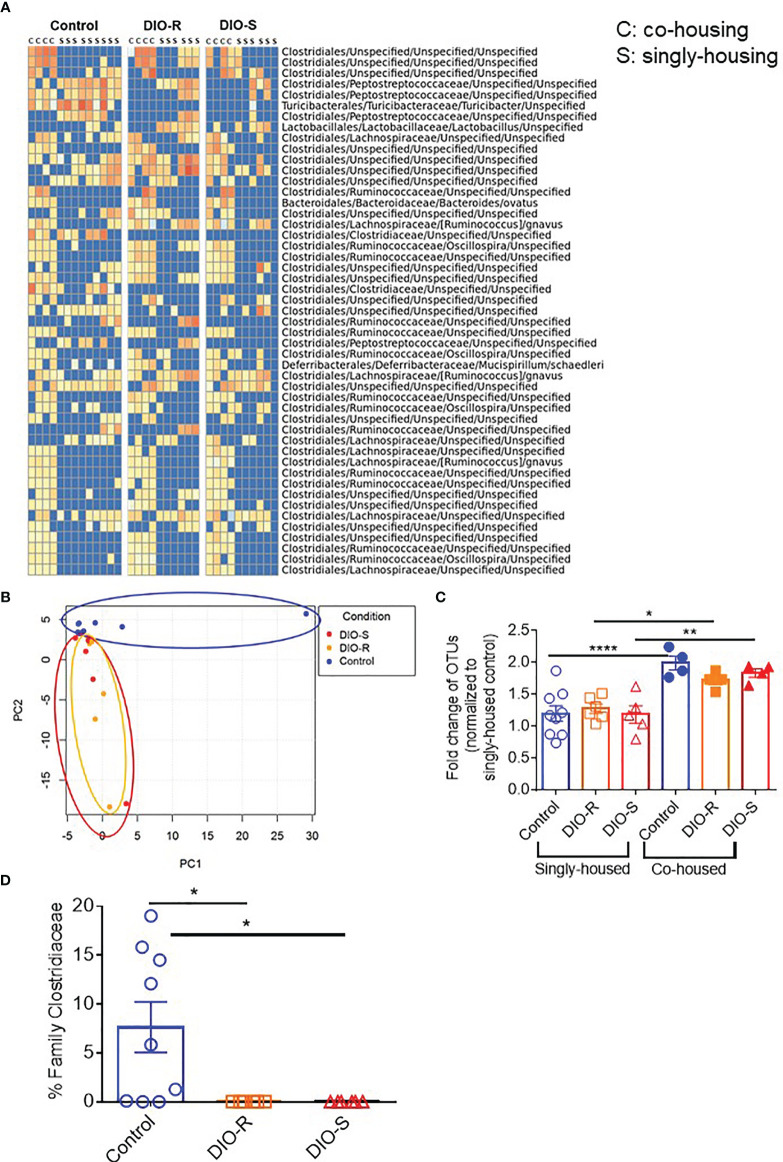
**(A)** Taxonomic profiles of resting control, DIO-R, and DIO-S BALB/c mice being co-housed or singly housed for 4 months (n=4-9/group). **(B)** Principle component analysis (PCA) of microbiome profiles of singly-housed control, DIO-R, and DIO-S BALB/c mice (n=6-9/group). **(C)** Fold change of OTUs in singly-housed versus co-housed control, DIO-R, and DIO-S BALB/c mice (n=4-9/group). **(D)** Abundance of family Clostridiaceae of singly-housed control, DIO-R, and DIO-S BALB/c mice (n=6-9/group). Bar graphs depict mean ±s.e.m. One-way ANOVA test was used in **(C, D)**. *p < 0.05, **p < 0.01, ****p < 0.0001.

We then characterized the microbiome status of the singly housed control, DIO-R, and DIO-S mice, as there would be no normalization as seen in the group-housed cohorts. It has been previously reported that cold temperature can markedly reduce microbiome diversity in mice, and the stresses of social isolation are likely responsible for the effects seen in single-housed cohorts (27). Our data showed that the taxon abundances in all three groups (single-housed control, DIO-R, and DIO-S) were very different when compared to their co-housed counterparts, yet DIO-R and DIO-S were similar to each other (**Figure 6A**). Principal component analysis revealed that the microbiome profiles of singly housed DIO-S and DIO-R mice were distributed in a scattered pattern (**Figure 6B**). Surprisingly, in contrast to the marked effect that mice on HF diets had on the microbiome compared to control diet mice, we observed similar microbiome alterations occurring between the microbiome of singly housed DIO-R, DIO-S, and control mice displaying similar reductions in diversity. Diversity assessment showed that all the singly housed mice had more restricted microbiome diversity compared to the co-housed mice, even more so than of that group-housed HF diet-fed mice (**Figure 6C**). However, because the singly housed control diet mice did not show evidence of the acute GVHD seen in the HF mice but also had significantly lower operational taxonomic units (OTUs) compared to group-housed control, these results suggest that microbiome diversity alone might not be a crucial indicator for acute GVHD outcomes in the absence of obesity. Interestingly, abundance of family Clostridiaceae in singly housed mice still stayed undetectable compared to high mean levels observed in the control mice (**Figure 6D**). These data suggest that HF diet consumption and fat content might play a more important role than simply microbiome alterations in order to induce the acute gut GVHD pathogenesis in mice.

## Discussion

To our knowledge, our results are the first to delineate the roles of body weight *vs.* HF diet exposure in conjunction with the microbiome on acute GVHD processes. We used a strain combination for allo-HSCT in which only HF diet and obesity induce acute gut GVHD, where, normally, chronic skin GVHD would result. We also took advantage of DIO resistance of BALB/c mice for distinguishing the role of adiposity *vs.* the diet itself on GVHD induction. Furthermore, the use of microbiome assessment involving group-housed *vs.* single-housed cohorts allowed for delineation of reductions in microbiome diversity in the development of acute GVHD in these mice due to the effects of single-housing stress in inducing microbiome alterations in mice. These results indicate that adiposity itself is the primary driver for the acute gut GVHD pathogenesis, while the diet itself does appear to exert some effects, as reflected in the survival and pathology observed. In contrast, using single-housed control mice on low-fat diet, where microbiome diversity reductions still occurred, had no effect on acute GVHD induction, indicating that while the microbiome alterations can indeed play a role in perhaps fueling ongoing acute GVHD processes, these alone are not sufficient for its induction in this strain combination. This is in agreement with our recent study in which prophylactic antibiotics could only partially ameliorate acute GVHD in DIO recipients (4) but perhaps surprising that obesity itself was the primary driver for induction of gut GVHD processes perhaps due to increased gut damage resulting from the cytoreductive conditioning in obese mice.

With the increasing incidence of obesity in the US and other countries in the world, as well as increased consumption of foods with HF content, our study raises salient points of how the metabolically unhealthy body condition critically contributes to poor overall survival and GVHD outcomes in the settings of allo-HSCT particularly impacting the gastrointestinal system. There are multiple factors that potentially play major roles in inducing acute gut GVHD in recipients with prolonged HF diet consumption and body fat deposition including, but not limited to, increased damage by cytoreductive conditioning and production of pro-inflammatory cytokines after radiation and HSCT exacerbating cytokine storm (4); the impaired intestinal barrier and integrity accommodate bacterial translocation to the bloodstream, causing systemic sepsis (4); the activation of T cells due to increased fatty acid metabolism (15, 28); the activation of antigen-presenting cells, i.e., dendritic cells, in the mesenteric lymph nodes that in turn recruit and further activate donor T cells to trigger greater tissue damage and inflammation (29, 30); and the reduced diversity of microbiota and taxa alterations that likely all contribute for acute gut GVHD pathogenesis.

HF diets have been shown to induce dysbiosis in the gut flora that contributes to low-grade inflammation, impaired antimicrobial peptide production, decreased tight junction protein expression, and mucus layer destruction (8, 9, 25, 31). These changes can lead to bacterial translocation from the intestine into the bloodstream, Toll-like receptor-mediated inflammation due to the release of pathogen-associated molecular patterns (PAMPs) (i.e., endotoxin, LPS, flagellin) or metabolites (i.e., bile acid) (10) that can facilitate more pro-inflammatory cytokine production, intestinal barrier deregulation, and continued GVHD-mediated gut pathology.

In assessing the complex role of the microbiome in GVHD, attention has centered on roles of certain bacterial flora or strains and the impact of reduced microbial diversity that has been well-documented to occur in obesity (4, 8, 9, 25). The results indicate that reductions alone in microbiome diversity cannot induce acute GVHD in this strain combination, although it is likely that it augments the impact of obesity and diet since antibiotics have been demonstrated to partially protect these DIO recipients (4). Similarly, the reduced GVHD occurring in the DIO-R mice suggests that diet can also play a role, although more detailed assessment of adiposity is needed to rule out that the DIO-R mice, while comparable in body weight to low-fat diet-fed mice, still have modest increases in adiposity that could be sufficient for the effects observed. All of the results presented would indicate that body fat alone and neither diet nor microbiome diversity is sufficient for the increased lethal acute gut GVHD observed. These data are also in agreement with studies demonstrating mice placed on HF diets for shorter periods of time in which body weight has not been significantly altered yet microbiome alterations have occurred, but no GVHD effects resulted (4). As the restricted microbiome diversity observed in single-housed mice regardless of diet is likely due to stress, it will be important to carefully delineate the impact of stress on microbiome composition, as it may also have a bearing on GVHD outcome. The results presented therefore demonstrate the critical role of body fat deposition on acute gut GVHD induction over that of diet and microbiome alterations.

## Data Availability Statement

The datasets presented in this study can be found in online repositories. The names of the repository/repositories and accession number(s) can be found below: http://www.ncbi.nlm.nih.gov/bioproject/758120, PRJNA758120.

## Ethics Statement

The animal study was reviewed and approved by the University of California, Davis, Institutional Animal Care and Use Committee (IACUC) standards.

## Author Contributions

LK and LV designed and performed experiments, analyzed results, and co-wrote the article. CD performed experiments and edited the article. EC performed histology scores and edited the article. LK, AM, and EM performed microbiome data analysis. EM, AM, and BB edited the article. WM directed the project, designed experiments, interpreted results, and co-wrote the article. All authors contributed to the article and approved the submitted version.

## Funding

This work was funded by NIH R01 CA214048 (WM), NIH R37 AI34495, HL56067 (BB), and the UC Davis Comprehensive Cancer Center Support Grant (CCSG) (P30 CA093373).

## Author Disclaimer

The content of this publication does not necessarily reflect the views or policies of the Department of Health and Human Services, nor does mention of trade names, commercial products, or organizations imply endorsement by the US Government.

## Conflict of Interest

BB receives remuneration as an advisor to Magenta Therapeutics and BlueRock Therapeutics; Research funding from BlueRock Therapeutics, Rheos Medicines, Equilibre biopharmaceuticals, Carisma Therapeutics, Inc., and is a co-founder of Tmunity Therapeutics.

AM has advisory role or research funding from Merck, Genentech, BMS, Incyte, Trisalus, MultiplexThera, EMD serono, Transgene.

The remaining authors declare that the research was conducted in the absence of any commercial or financial relationships that could be construed as a potential conflict of interest.

## Publisher’s Note

All claims expressed in this article are solely those of the authors and do not necessarily represent those of their affiliated organizations, or those of the publisher, the editors and the reviewers. Any product that may be evaluated in this article, or claim that may be made by its manufacturer, is not guaranteed or endorsed by the publisher.

## Supplementary Material

Supplementary Figure 1
**(A)** Representative images of control, DIO-R, and DIO-S mice. **(B)** Kinetics of body weight gain of BALB/c mice on LF or HF diet. **(C)** Acute GVHD clinical scores post-HSCT (n=12/group). **(D)** Body weights (g) of co-housed control, DIO-R, and DIO-S post-HSCT. **(E)** Quantification of gene expression of caspase-3 in the ileum of control, DIO-R, and DIO-S mice at 48 hours after radiation (n=3/group). Graphs depict mean ±s.e.m. Body weght curve **(B)** and clinical scores **(C)** were analyzed by 2-way analysis of variance (ANOVA) with Tukey's post hoc test for comparison among groups. Unpaired Student’s t test was used in **(E)**. *p < 0.05, **p < 0.01, ****p < 0.0001, NS, not significant.

Supplementary Figure 2
**(A)** Flow cytometry gating strategy on activated dendritic cells in the mesenteric lymph nodes 4 days after HSCT. **(B)** Quantification of numbers of activated dendritic cells in the mesenteric lymph nodes 4 days after HSCT (n=4/group). Graphs depict mean ±s.e.m. One-way ANOVA test was used in **(B)**. *p < 0.05, **p < 0.01.

## References

[1] de Heredia FP Gomez-Martinez S Marcos A. Obesity, Inflammation and the Immune System. Proc Nutr Soc (2012) 71(2):332–8. doi: 10.1017/S0029665112000092 22429824

[2] Milner JJ Beck MA. The Impact of Obesity on the Immune Response to Infection. Proc Nutr Soc (2012) 71(2):298–306. doi: 10.1017/S0029665112000158 22414338 PMC4791086

[3] Mirsoian A Bouchlaka MN Sckisel GD Chen M Pai CC Maverakis E. Adiposity Induces Lethal Cytokine Storm After Systemic Administration of Stimulatory Immunotherapy Regimens in Aged Mice. J Exp Med (2014) 211(12):2373–83. doi: 10.1084/jem.20140116 PMC4235633 25366964

[4] Khuat LT Le CT Pai CS Shields-Cutler RR Holtan SG Rashidi A. Obesity Induces Gut Microbiota Alterations and Augments Acute Graft-*Versus*-Host Disease After Allogeneic Stem Cell Transplantation. Sci Transl Med (2020) 12(571):eaay7713. doi: 10.1126/scitranslmed.aay7713 33239390 PMC8525601

[5] Navarro WH Loberiza FR Jr. Bajorunaite R van Besien K Vose JM Lazarus HM. Effect of Body Mass Index on Mortality of Patients With Lymphoma Undergoing Autologous Hematopoietic Cell Transplantation. Biol Blood Marrow Transplant (2006) 12(5):541–51. doi: 10.1016/j.bbmt.2005.12.033 16635789

[6] Fuji S Kim SW Yoshimura K Akiyama H Okamoto S Sao H. Possible Association Between Obesity and Posttransplantation Complications Including Infectious Diseases and Acute Graft-*Versus*-Host Disease. Biol Blood Marrow Transplant (2009) 15(1):73–82. doi: 10.1016/j.bbmt.2008.10.029 19135945

[7] Pereira AZ de Almeida-Pitito B Eugenio GC Ruscitto do Prado R Silva CC Hamerschlak N. Impact of Obesity and Visceral Fat on Mortality in Hematopoietic Stem Cell Transplantation. JPEN J Parenter Enteral Nutr (2020). doi: 10.1002/jpen.2048 33236392

[8] Boudry G Hamilton MK Chichlowski M Wickramasinghe S Barile D Kalanetra KM. Bovine Milk Oligosaccharides Decrease Gut Permeability and Improve Inflammation and Microbial Dysbiosis in Diet-Induced Obese Mice. J Dairy Sci (2017) 100(4):2471–81. doi: 10.3168/jds.2016-11890 PMC5481169 28131576

[9] Hamilton MK Ronveaux CC Rust BM Newman JW Hawley M Barile D. Prebiotic Milk Oligosaccharides Prevent Development of Obese Phenotype, Impairment of Gut Permeability, and Microbial Dysbiosis in High Fat-Fed Mice. Am J Physiol Gastrointest Liver Physiol (2017) 312(5):G474–87. doi: 10.1152/ajpgi.00427.2016 PMC5451559 28280143

[10] Murakami Y Tanabe S Suzuki T. High-Fat Diet-Induced Intestinal Hyperpermeability is Associated With Increased Bile Acids in the Large Intestine of Mice. J Food Sci (2016) 81(1):H216–222. doi: 10.1111/1750-3841.13166 26595891

[11] Jenq RR Ubeda C Taur Y Menezes CC Khanin R Dudakov JA. Regulation of Intestinal Inflammation by Microbiota Following Allogeneic Bone Marrow Transplantation. J Exp Med (2012) 209(5):903–11. doi: 10.1084/jem.20112408 PMC3348096 22547653

[12] Jenq RR Taur Y Devlin SM Ponce DM Goldberg JD Ahr KF. Intestinal Blautia Is Associated With Reduced Death From Graft-*Versus*-Host Disease. Biol Blood Marrow Transplant (2015) 21(8):1373–83. doi: 10.1016/j.bbmt.2015.04.016 PMC4516127 25977230

[13] Mathewson ND Jenq R Mathew AV Koenigsknecht M Hanash A Toubai T. Gut Microbiome-Derived Metabolites Modulate Intestinal Epithelial Cell Damage and Mitigate Graft-*Versus*-Host Disease. Nat Immunol (2016) 17(5):505–13. doi: 10.1038/ni.3400 PMC4836986 26998764

[14] Taur Y Jenq RR Perales MA Littmann ER Morjaria S Ling L. The Effects of Intestinal Tract Bacterial Diversity on Mortality Following Allogeneic Hematopoietic Stem Cell Transplantation. Blood (2014) 124(7):1174–82. doi: 10.1182/blood-2014-02-554725 PMC4133489 24939656

[15] Byersdorfer CA Tkachev V Opipari AW Goodell S Swanson J Sandquist S. Effector T Cells Require Fatty Acid Metabolism During Murine Graft-*Versus*-Host Disease. Blood (2013) 122(18):3230–7. doi: 10.1182/blood-2013-04-495515 PMC3814737 24046012

[16] Rebeles J Green WD Alwarawrah Y Nichols AG Eisner W Danzaki K. Obesity-Induced Changes in T-Cell Metabolism Are Associated With Impaired Memory T-Cell Response to Influenza and Are Not Reversed With Weight Loss. J Infect Dis (2019) 219(10):1652–61. doi: 10.1093/infdis/jiy700 PMC6473176 30535161

[17] James BR Tomanek-Chalkley A Askeland EJ Kucaba T Griffith TS Norian LA. Diet-Induced Obesity Alters Dendritic Cell Function in the Presence and Absence of Tumor Growth. J Immunol (2012) 189(3):1311–21. doi: 10.4049/jimmunol.1100587 PMC3401274 22745381

[18] Boi SK Buchta CM Pearson NA Francis MB Meyerholz DK Grobe JL. Obesity Alters Immune and Metabolic Profiles: New Insight From Obese-Resistant Mice on High-Fat Diet. Obes (Silver Spring) (2016) 24(10):2140–9. doi: 10.1002/oby.21620 PMC5039085 27515998

[19] Pai CC Chen M Mirsoian A Grossenbacher SK Tellez J Ames E. Treatment of Chronic Graft-*Versus*-Host Disease With Bortezomib. Blood (2014) 124(10):1677–88. doi: 10.1182/blood-2014-02-554279 PMC4155274 25009225

[20] Pai CS Khuat LT Chen M Murphy WJ Abedi M. Therapeutic Effects of a NEDD8-Activating Enzyme Inhibitor, Pevonedistat, on Sclerodermatous Graft-*Versus*-Host Disease in Mice. Biol Blood Marrow Transplant (2017) 23(1):30–7. doi: 10.1016/j.bbmt.2016.10.022 PMC5469294 27815049

[21] Cooke KR Kobzik L Martin TR Brewer J Delmonte J Jr Crawford JM. An Experimental Model of Idiopathic Pneumonia Syndrome After Bone Marrow Transplantation: I. Roles Minor H Antigens endotoxin. Blood (1996) 88(8):3230–9. doi: 10.1182/blood.V88.8.3230.bloodjournal8883230 8963063

[22] Segata N Izard J Waldron L Gevers D Miropolsky L Garrett WS. Metagenomic Biomarker Discovery and Explanation. Genome Biol (2011) 12(6):R60. doi: 10.1186/gb-2011-12-6-r60 21702898 PMC3218848

[23] Hamilton BL. L3T4-Positive T Cells Participate in the Induction of Graft-vs-Host Disease in Response to Minor Histocompatibility Antigens. J Immunol (1987) 139(8):2511–5. 3498761

[24] Schroeder MA DiPersio JF. Mouse Models of Graft-*Versus*-Host Disease: Advances and Limitations. Dis Model Mech (2011) 4(3):318–33. doi: 10.1242/dmm.006668 PMC3097454 21558065

[25] Ley RE Backhed F Turnbaugh P Lozupone CA Knight RD Gordon JI. Obesity Alters Gut Microbial Ecology. Proc Natl Acad Sci USA (2005) 102(31):11070–5. doi: 10.1073/pnas.0504978102 PMC1176910 16033867

[26] Ericsson AC Franklin CL. Manipulating the Gut Microbiota: Methods and Challenges. ILAR J (2015) 56(2):205–17. doi: 10.1093/ilar/ilv021 PMC4554251 26323630

[27] Worthmann A John C Ruhlemann MC Baguhl M Heinsen FA Schaltenberg N. Cold-Induced Conversion of Cholesterol to Bile Acids in Mice Shapes the Gut Microbiome and Promotes Adaptive Thermogenesis. Nat Med (2017) 23(7):839–49. doi: 10.1038/nm.4357 28604703

[28] Sikder K Shukla SK Patel N Singh H Rafiq K. High Fat Diet Upregulates Fatty Acid Oxidation and Ketogenesis via Intervention of PPAR-Gamma. Cell Physiol Biochem (2018) 48(3):1317–31. doi: 10.1159/000492091 PMC6179152 30048968

[29] Chen Y Tian J Tian X Tang X Rui K Tong J. Adipose Tissue Dendritic Cells Enhances Inflammation by Prompting the Generation of Th17 Cells. PloS One (2014) 9(3):e92450. doi: 10.1371/journal.pone.0092450 24642966 PMC3958510

[30] Cheng L Jin H Qiang Y Wu S Yan C Han M. High Fat Diet Exacerbates Dextran Sulfate Sodium Induced Colitis Through Disturbing Mucosal Dendritic Cell Homeostasis. Int Immunopharmacol (2016) 40:1–10. doi: 10.1016/j.intimp.2016.08.018 27567245

[31] Ahmad R Rah B Bastola D Dhawan P Singh AB. Obesity-Induces Organ and Tissue Specific Tight Junction Restructuring and Barrier Deregulation by Claudin Switching. Sci Rep (2017) 7(1):5125. doi: 10.1038/s41598-017-04989-8 28698546 PMC5505957

