# Supplementary figures and images for: Mechanisms by Which Obesity Promotes Acute Graft-Versus-Host Disease in Mice
Source: Front Immunol. 2021 Oct 11;12:752484. doi: 10.3389/fimmu.2021.752484 (PMC8542879; doi:10.3389/fimmu.2021.752484)

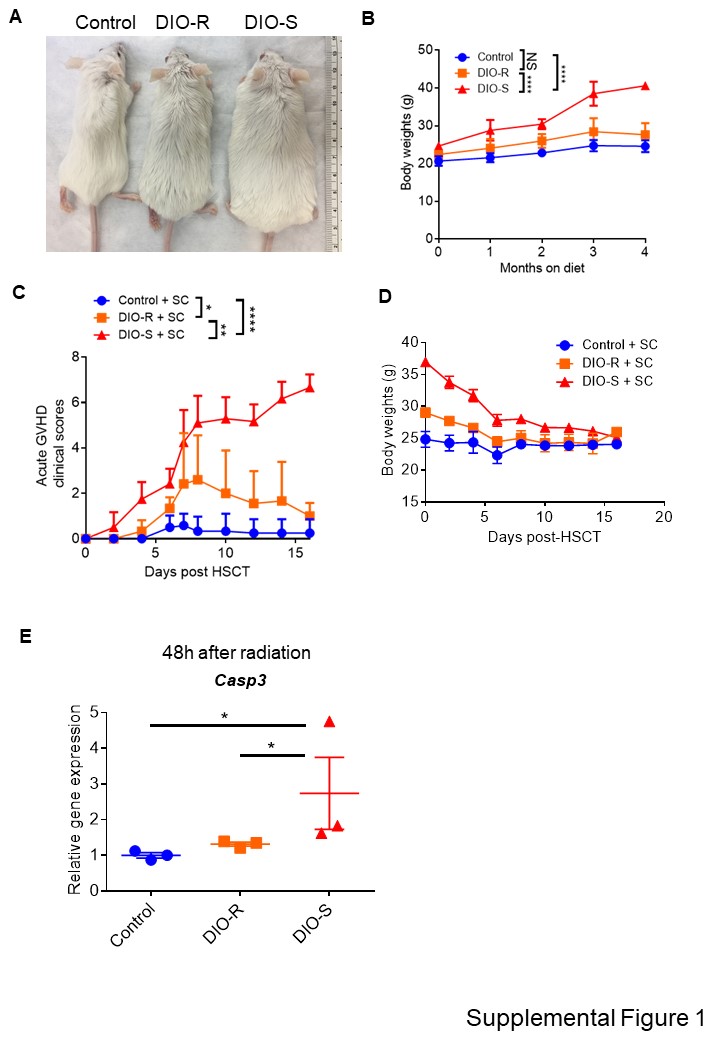

Supplement: Supplementary Figure 1 — (A) Representative images of control, DIO-R, and DIO-S mice. (B) Kinetics of body weight gain of BALB/c mice on LF or HF diet. (C) Acute GVHD clinical scores post-HSCT (n=12/group). (D) Body weights (g) of co-housed control, DIO-R, and DIO-S post-HSCT. (E) Quantification of gene expression of caspase-3 in the ileum of control, DIO-R, and DIO-S mice at 48 hours after radiation (n=3/group). Graphs depict mean ±s.e.m. Body weght curve (B) and clinical scores (C) were analyzed by 2-way analysis of variance (ANOVA) with Tukey's post hoc test for comparison among groups. Unpaired Student’s t test was used in (E). *p < 0.05, **p < 0.01, ****p < 0.0001, NS, not significant. [file Image_1.jpeg]

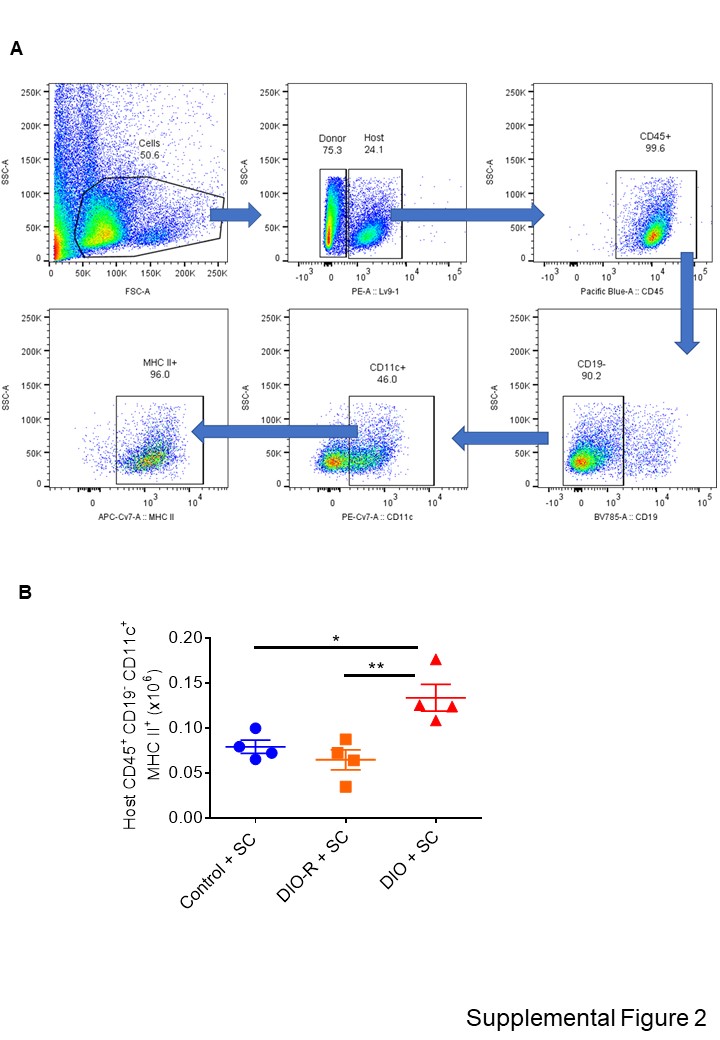

Supplement: Supplementary Figure 2 — (A) Flow cytometry gating strategy on activated dendritic cells in the mesenteric lymph nodes 4 days after HSCT. (B) Quantification of numbers of activated dendritic cells in the mesenteric lymph nodes 4 days after HSCT (n=4/group). Graphs depict mean ±s.e.m. One-way ANOVA test was used in (B). *p < 0.05, **p < 0.01. [file Image_2.jpeg]
